# Revised and Improved Procedure for Immunolocalization of Male Meiotic Chromosomal Proteins and Spindle in Plants without the Use of Enzymes

**DOI:** 10.3390/plants7040093

**Published:** 2018-10-29

**Authors:** Kuntal De, Li Yuan, Christopher A. Makaroff

**Affiliations:** 1 Department of Radiation Oncology, James Cancer Hospital and Comprehensive Cancer Center, The Ohio State University Wexner School of Medicine, Columbus, OH 43210, USA; 2 State Key Laboratory of Crop Stress Biology for Arid areas, College of Horticulture, Northwest A&F University, Yangling 712100, China; lyuan@nwafu.edu.cn; 3 Hughes Laboratories, Department of Chemistry and Biochemistry, Miami University, Oxford, OH 45056, USA; makaroca@miamioh.edu

**Keywords:** Arabidopsis, spindle, synapsis, cohesin, meiosis, immunofluorescence

## Abstract

Immunolocalization studies to visualize the distribution of proteins on meiotic chromosomes have become an integral part of studies on meiosis in the model organism *Arabidopsis thaliana*. These techniques have been used to visualize a wide range of meiotic proteins involved in different aspects of meiosis, including sister chromatid cohesion, recombination, synapsis, and chromosome segregation. However, the analysis of meiotic spindle structure by immunofluorescence is of outstanding importance in plant reproductive biology and is very challenging. In the following report, we describe the complete and easy protocol for the localization of proteins to the male meiotic spindle and male meiotic chromosomes. The protocol is fast, improved, and robust without the use of any harsh enzymes.

## 1. Background

In plant biology, immunofluorescence is crucial for a variety of purposes, including protein localization and protein–protein interaction. The main challenge is the presence of cell wall, which makes the specimens thick. Plant cells suffer from poor permeabilization of fluorescent dyes. Rigorous enzyme treatment is necessary, which improves the penetration of antibodies to the inside layers of the cell. Presently, immunolocalization of chromosomal proteins consists of numerous steps, is poorly reproducible due to limitation of antibody exposure to the deeper cells, and furthers the status of tissue preservation after the administration of harsh enzymatic chemicals. In this paper, we have set precise parameters for bud dissection and fixation and developed a protocol for reproducible visualization of spindle proteins during plant meiosis.

*Arabidopsis thaliana* is a model organism to study meiosis because of its small genome. Cytological experiments on Arabidopsis are very difficult to analyze at a molecular level. A number of different techniques have been developed throughout the years to analyze meiotic chromosomes in Arabidopsis. We routinely use paraformaldehyde fixation, which preserves the cellular and chromosome structure properly as it crosslinks proteins intermolecularly. After paraformaldehyde fixation, a number of steps are followed, including cell permeabilization, flash freezing, squashing by physical force, enzyme digestion, and detergent treatment.

However, one of the important microscopic studies that seem to be challenging is the analysis of spindle structure on meiotic chromosomes. We have rigorously developed a reliable, easy and fast protocol specifically for spindle structure in plants. The reported protocol allows robust immunolabeling of spindle fibers in *Arabidopsis thaliana* meiocytes, which thus enables higher resolution independent of the treatment of harsh enzymes. We have also taken the advantage of this protocol to stain male meiotic chromosomal proteins, including a cohesin protein SYN1, a transverse filament protein of synaptonemal complex ZYP1, and axial/lateral filament proteins of chromosomal axes, ASY1. The protocol is the first-ever study to report the immunolocalization of meiotic chromosomal proteins without the use of enzymes.

## 2. Method

**Figure 1:**
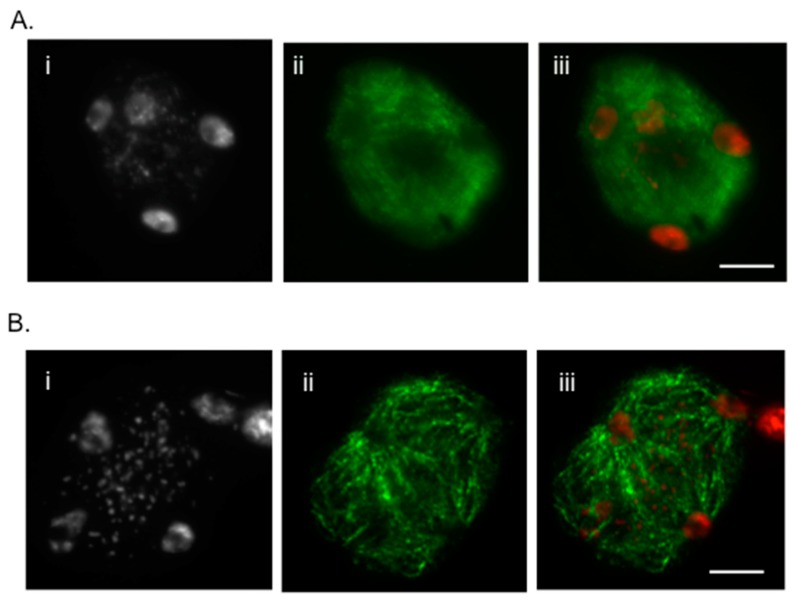
(**A**) Immunolocalization of beta tubulin in paraformaldehyde-fixed male meiocytes with enzymes. (i) 4’,6-diaminido-2-phenlyinidole (DAPI)-stained tetrad, (ii) beta tubulin stained tetrad and (iii) Merged. Chromosomes are counterstained with DAPI (left image of each panel); Bar = 10 μm. (**B**) Immunolocalization of beta tubulin in paraformaldehyde-fixed male meiocytes without enzymes. (i) DAPI-stained Tetrad, (ii) beta tubulin stained tetrad and (iii) Merged. Chromosomes are counterstained with DAPI (left image of each panel); Bar = 10 μm.

**Figure 2:**
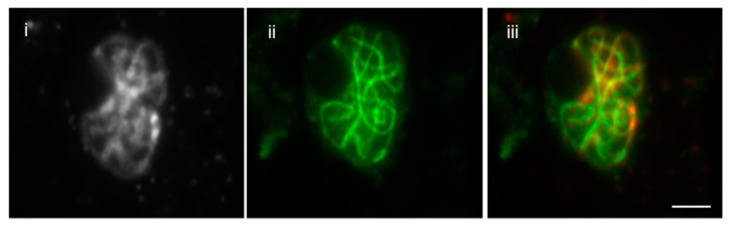
Immunolocalization of SYN1 in paraformaldehyde-fixed male meiocytes without enzymes. (i) DAPI-stained pachytene chromosome, (ii) SYN1 stained pachytene chromosome and (iii) Merged. Chromosomes are counterstained with DAPI (left image of each panel); Bar = 10 μm.

**Figure 3:**
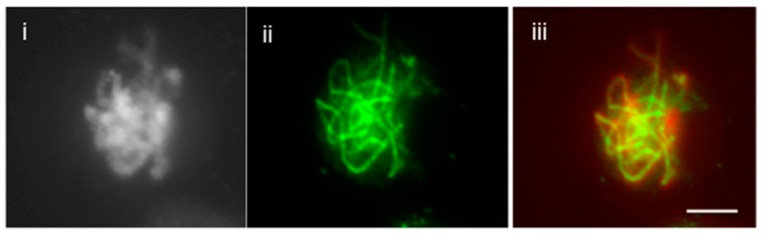
Immunolocalization of ZYP1 in paraformaldehyde-fixed male meiocytes without enzymes. (i) DAPI-stained pachytene chromosome, (ii) ZYP1 stained pachytene chromosome and (iii) Merged. Chromosomes are counterstained with DAPI (left image of each panel); Bar = 10 μm.

**Figure 4:**
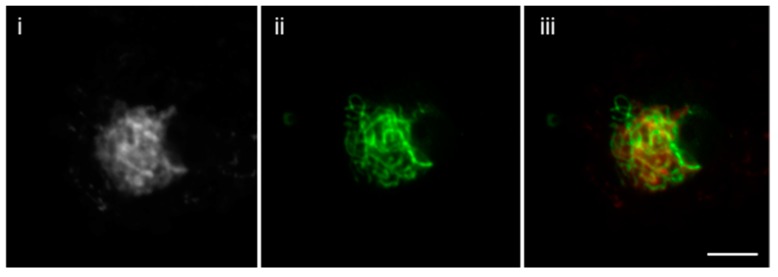
Immunolocalization of ASY1 in paraformaldehyde-fixed male meiocytes without enzymes. (i) DAPI-stained pachytene chromosome, (ii) ASY1 stained stained pachytene chromosome and (iii) Merged. Chromosomes are counterstained with DAPI (left image of each panel); Bar = 10 μm.

**Figure 5:**
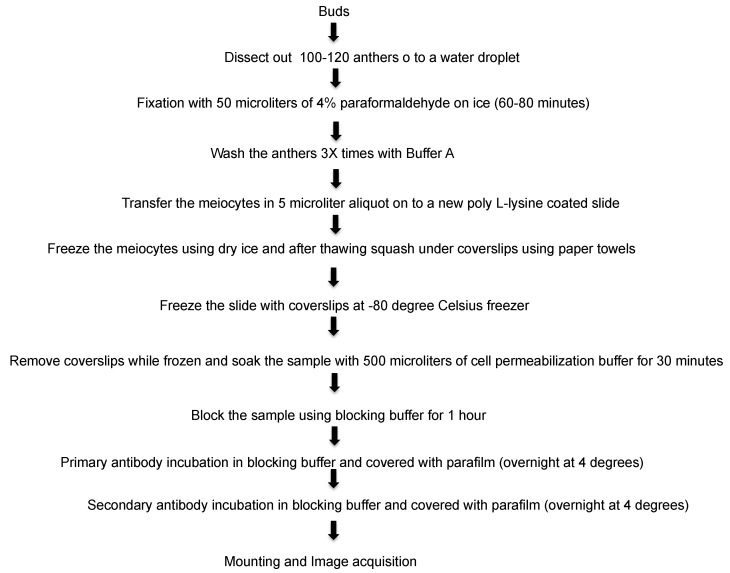
Complete steps for the meiotic chromosomal proteins and spindle immunolocalization technique.

This method is improved and revised from a previous protocol [1]. Here, we report the much simplified method to attain fast and reliable immunolocalization specifically for meiotic chromosomes and spindle structure. It is well known that the immunolocalization of spindle fibers is very difficult and requires careful execution to attain reproducible results. To achieve that, we developed an easy technique that does not require the use of enzymes. With mechanical breaking of the cells and releasing meiocytes, the structure of chromosome is determined in this technique. The difference in the immunolocalization technique with or without enzymes is shown in Figure 1A,B. Without the use of enzymes, the spindle fibers are more precisely distinguishable, whereas with the use of enzymes, the fibers are overlapped between each other. Previous studies from our lab have demonstrated that localization SYN1 cohesin protein localizes to arms of meiotic chromosomes from approximately meiotic interphase to anaphase I [2]. We were also successful in determining localization of SYN1 cohesin protein on meiotic chromosomes. During pachytene, SYN1 protein lined the chromosomes (Figure 2). To validate that the protocol can be used universally for other meiotic chromosomal proteins, we probed for a couple of proteins, which included ZYP1 and ASY1. ZYP1, an axial element protein for chromosomes, appears at zygotene as foci. ZYP1 signals extend during pachytene, producing a continuous signal between the synapsed homologous chromosomes [3]. Using this protocol, we were able to see clean ZYP1 signal, as shown in Figure 3. Additionally, the protocol was used to investigate the distribution of ASY1. ASY1 is a meiotic chromosomal protein that associates with axial and lateral elements during prophase I in *Arabidopsis thaliana* [4]. We were successful in visualizing ASY1 signals, as shown in Figure 4. We have also reported an overview of the main steps of the procedure in Figure 5 The whole procedure is described step-by-step below with important comments whenever needed.

**Revised protocol**

1. Under a dissecting microscope, place 100 microlitres (μL) of water on a Labtek slide. Dissect 100–120 anthers (on to the water droplet).
  Comments: To remove water or any other solution, use a fine-tip needle.
2. Transfer 50 μL of 4% paraformaldehyde fixative solution onto the dissected anthers.
3. Fix the anthers for 60–80 min on ice.
4. Wash the anthers three times, 5 min each, with Buffer A.
  Comments: After three washes, aggregate the anthers and quickly add water. Aspirate the water with the help of syringe. Repeat this step two more times (5 min each). Remember to keep 5 μL of water with the sample during each wash to prevent it from drying.
5. Remove the wash buffer and add 10–20 μL of water to the anthers. Cover the anthers with a coverslip.
6. Gently press on the coverslip using the tip of the fine forceps to release the meiocytes. Make sure the coverslips do not break.
  Comment: Hard press will break the coverslip, which will be critical to collect the released meiocytes.
7. Add 5–10 μL of water to one side of the coverslip and collect the solution that contains meiocytes from the other side of the coverslip using a fine pipette tip.
8. Transfer the meiocytes onto a new poly-L-lysine-coated slide and incubate on dry ice for 10–15 min.
9. Thaw the slide to room temperature.
10. Transfer 5 μL of the meiocyte solution onto each of the 5–10 new coated slides and cover the cells with a coverslip.
11. Set the slides with coverslips precisely under folder paper towel and squash the sample area with the thumb. This step should be deliberately performed as increasing pressure has a propensity to break the chromosomes.
12. Freeze the slide at −80 °C freezer with coverslips hanging outside the edge of the slide.
13. Remove the coverslip while still frozen and dry the slides at room temperature.

Cell Permeabilization

From this step, we have omitted several steps from a previously published protocol [1] for optimizing results when immunolocalizing spindle proteins and chromosomal proteins. Our new steps for cell permeabilization are as follows:

1. Soak the sample with 500 μL of cell permeabilization solution at room temperature with/without agitation for 30 min.
2. Wash slides twice with 500 μL of wash buffer for 5 min each wash.

Immunolocalization Procedure

1. Block the sample in 200 μL of blocking buffer for 1 h.
2. Dilute primary antibody (to 1000-fold) in blocking buffer.
3. Apply 100 μL of diluted antibody to the sample area and put parafilm.
4. Incubate the slides in a slide incubation box at 4 °C overnight.
5. Next day, wash thrice with wash buffer and make sure the slides do not dry out.
6. Apply the secondary antibody (to 1000-fold) diluted in blocking solution to the sample area and cover it with parafilm.
7. Incubate overnight at 4 °C in the slide incubation box.
8. Wash the secondary antibody with wash buffer three times and semidry the slides.
9. Apply 5 μL of 4’,6-diaminido-2-phenlyinidole (DAPI) in Vectashield on the sample area and put coverslip for image acquisition.

## 3. Materials

### 3.1. Plants

1. Arabidopsis seeds should be sown in pots containing a commercial soil mix, such as Metro-Mix360 (SunGro Horticulture, Vancouver, BC, Canada). Grow plants in a plant growth chamber at 23 °C with a 16 h light/8 h dark cycle. Collect inflorescences from young, healthy *Arabidopsis thaliana* plants.

### 3.2. Tools and Equipment

1. Dry ice and −80 °C refrigerator
2. Coverslips and poly-L-lysine-coated slides
3. Slide incubation box with a wet paper towel to keep proper humidity
4. Dumont Forceps with long, fine tips
5. Commercially available water repellent slides, such as the Lab-Tek™ Chamber Slide™ System (Thermo Fisher Scientific, Waltham, MA, USA)
6. Stereomicroscope, florescence microscope, and image acquisition software

### 3.3. Solutions Recipe

Paraformaldehyde (4%) fixative in buffer A: 4 g of paraformaldehyde in 100 mL of buffer A containing 0.01% Triton X-100. Heat at 55 °C for up to 2 h with occasional mixing or until the solution becomes clear.

Slide coating solution: Add 50 μL of 0.1% (w/v) poly-L-lysine (Sigma Aldrich, St. Louis, MO, USA) to 50 mL of deionized water. Alternatively, use a mix of 20 μL of 3-methacryloxypropyl-trimethoxysilane in 50 mL of 95% ethanol, 0.5% glacial acid.

Buffer A: 15 mM PIPES, 80 mM KCl, 20 mM NaCl, 2 mM Ethylenediaminetetraacetic acid (EDTA), 0.5 mM ethylene glycol-bis (β-aminoethyl ether)-*N*,*N*,*N*′,*N*′-tetraacetic acid (EGTA), 0.5 mM spermidine, 0.15 mM spermine, 1 mM DTT, adjust pH to 5.8.

Wash buffer: 1× PBS, 0.1% Tween 20, 1 mM EDTA.

Cell wall permeabilization solution: mix 100 μL of Triton X-100 in 10 mL in washing buffer.

2% blocking buffer: Dissolve 0.2 g BSA in 10 mL of washing buffer.

Chromosome counterstain solution: DAPI in Vectashield (Vector Laboratory, Burlingame, CA, USA).

## 4. Conclusions

The protocol can be widely used in different plant species to immunolabel meiocytes as the harsh enzymatic interaction could change the conformation of the target protein and hence, there might be inhibition of antibody accessibility. The reported protocol preserves the native structure of the protein, and accurate protein localization can be studied.

## References

[1] Yang X., Yuan L., Makaroff C.A. (2013). Immunolocalization Protocols for Visualizing Meiotic Proteins in Arabidopsis thaliana: Method 3. Methods Mol. Biol..

[2] Cai X., Dong F., Edelmann R.E., Makaroff C.A. (2003). SYN1 cohesin protein is required for sister chromatid arm cohesion and homologous chromosome pairing. J. Cell Sci..

[3] Higgins J.D., Sanchez-Moran E., Armstrong S.J., Jones G.H., Franklin F.C.H. (2005). The Arabidopsis synaptonemal complex protein ZYP1 is required for chromosome synapsis and normal fidelity of crossing over. Genes Dev..

[4] Armstrong S.J., Caryl A.P., Jones G.H., Franklin F.C.H. (2002). Asy1, a protein required for meiotic chromosome synapsis, localizes to axis-associated chromatin in Arabidopsis and Brassica. J. Cell Sci..

